# A Presentation of Cerebritis Secondary to Granulomatosis with Polyangiitis (Wegener)

**DOI:** 10.1155/2014/914530

**Published:** 2014-05-13

**Authors:** James Norman, Ira Pande, Timothy Taylor, Bruno Gran

**Affiliations:** ^1^Department of Neurology, Nottingham University Hospitals NHS Trust, Queen's Medical Centre Campus, Nottingham NG7 2UH, UK; ^2^Department of Rheumatology, Nottingham University Hospitals NHS Trust, Queen's Medical Centre Campus, Nottingham NG7 2UH, UK; ^3^Department of Radiology, Nottingham University Hospitals NHS Trust, Queen's Medical Centre Campus, Nottingham NG7 2UH, UK; ^4^Clinical Neurology Research Group, Division of Clinical Neuroscience, University of Nottingham School of Medicine, C Floor South Block, Queen's Medical Centre, Nottingham NG7 2UH, UK

## Abstract

Neurological manifestations of GPA are common, most frequently as a peripheral neuropathy. Cerebritis as a principal presentation is extremely rare. We report a patient who presented with subacute progression of ataxia, confusion, and vacant episodes. An MRI of her brain showed bilateral signal abnormalities in the cingulate and superior sagittal gyrus while a staging CT revealed a mass in the right upper lobe of the patient's lung with a satellite nodule. C-ANCA antibodies specific for PR3 at high titres were positive and a diagnosis of GPA was made. The patient was commenced on intravenous methylprednisolone followed by cyclophosphamide and responded well to treatment. GPA is a rare and treatable differential diagnosis for confused patients with acute or subacute neurological features and unusual MRI findings.

## 1. Introduction

Granulomatosis with polyangiitis (GPA), formerly referred to as Wegener's granulomatosis [1], is a vasculitis of the small and medium sized vessels of unknown aetiology. It is strongly associated with cytoplasmic antineutrophil cytoplasmic antibodies (c-ANCA) with PR3 specificity [2] and can affect any organ, though it is particularly linked to the kidneys and respiratory tract. Neurological manifestations are common, affecting up to a third of patients. However, cerebritis is a very rare presentation, two reports of which are the definitive studies of neurological involvement in GPA by Nishino et al. [3, 4] (a fourth case excludes Wegener's granulomatosis as a differential when diagnosing cocaine-induced encephalocele [5]). This case report details a patient whose only presenting symptoms were secondary to cerebritis.

## 2. Case Report

A 64-year-old Caucasian woman presented to her local district general hospital with a subacute progression of ataxia, initially diagnosed in the community as labyrinthitis. Her past medical history was only notable for a thyroidectomy in 1993 for a papillary carcinoma, which was considered cured at subsequent follow-up. Her only regular medication was thyroxin and she had no allergies, occupational exposure to hazardous materials, or family history of note. She neither smoked nor drank alcohol.

**Figure 1:**
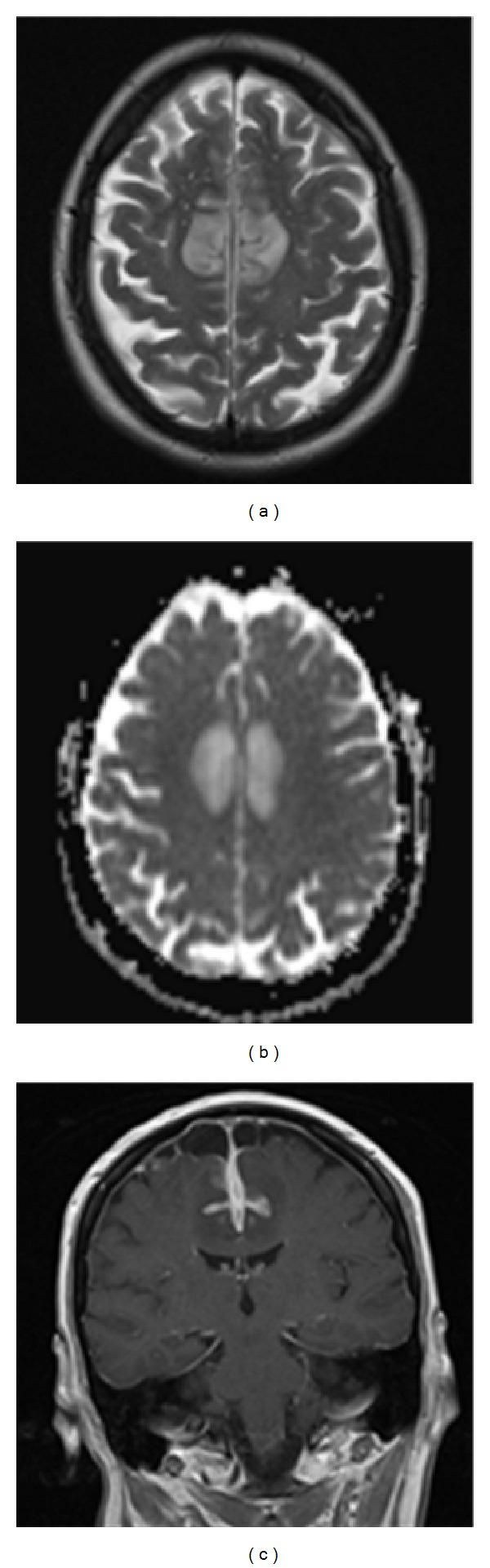
Imaging at presentation (08/2012). (a) Axial T2 image demonstrating moderate parafalcine oedema with local mass effect. (b) Axial apparent diffusion coefficient map confirming free water diffusion, that is, vasogenic oedema. (c) Coronal T1 postcontrast image demonstrating avid parafalcine leptomeningeal and cortical enhancement.

Upon transfer to our hospital, clinical examination demonstrated marked confusion, ataxia, and horizontal nystagmus; power, muscle tone, and deep tendon reflexes were normal. There were no significant chest, abdominal, or other physical findings and temperature was normal. An MRI of her brain showed bilateral parenchymal oedema and leptomeningeal/cortical enhancement associated with the cingulate and superior sagittal gyrus (Figure 1). A lumbar puncture revealed opening CSF pressure >34 cm H_2_O (the maximum value on the manometers used) with reactive cytology (although no white blood cells were detected upon automatic counting, cytospin analysis revealed lymphocytes and plasma cells together with macrophages and an occasional eosinophil; no neoplastic cells were observed) and raised CSF protein (742 mg/L). A staging CT revealed a mass in the right upper lobe of the patient's lung with a satellite nodule (image not shown) and a plan for CT guided biopsy was made. Serum angiotensin converting enzyme was normal and an electroencephalogram showed no epileptiform activity.

Detailed questioning by the rheumatologist elicited a history of two years of joint pain responsive to NSAIDs, two episodes of an inflammatory eye disease (likely episcleritis) treated successfully with topical steroids, and intermittent nose bleeds, though none recent. A provisional diagnosis of GPA was suggested, with differential diagnoses including infection (particularly tuberculosis) and cancer, possibly with a paraneoplastic syndrome. However, strongly positive c-ANCA specific for PR3 suggested a vasculitic condition and the patient was commenced on intravenous methylprednisolone, with an early good clinical response in terms of both limb power and improved higher cognitive function. In terms of differential diagnosis, the absence of elevated ACE and calcium, typical lung parenchymal changes, cranial neuropathies or seizures, signs of myelopathy, radiculopathy, peripheral neuropathy, or neuroendocrine dysfunction (hypothyroidism was due to previous thyroidectomy) did not support a diagnosis of sarcoidosis. Cerebrospinal fluid analysis did not show either acid-fast bacilli or growth of Mycobacteria; the lung abnormalities were not suggestive of tuberculosis. Meningeal carcinosis was unlikely in the absence of detectable systemic primary tumours, in particular, breast and melanoma (negative clinical examination) and lung (parenchymal lesions not suggestive of lung cancer). In addition, cerebrospinal fluid cytology did not show any cancerous cells.

Following the third dose of IV steroids, the patient became extremely confused and complained of chest pain, with clinical findings of tachycardia, tachypnea, hypotension, and desaturation on room air. Serial electrocardiograms demonstrated anterior ST depression and T wave inversion extending inferiorly. Troponin I was transiently elevated to 0.110 *μ*g/L. An arterial blood gas showed type 1 respiratory failure. The patient was transferred from the neurology ward to the coronary care unit, though the cardiology team were unconvinced that the ECG changes represented acute coronary syndrome. An urgent CTPA did not identify a pulmonary embolus, and coronary arteries were patent. However, interval cavitation in the right upper lobe mass lesion was demonstrated. A subsequent echocardiogram was also normal. These features in association with positivity for c-ANCA and the presence of anti-proteinase 3 antibodies at high titre (39 U/mL; reference 0–5 U/mL), alongside negative cultures for microorganisms of the patient's CSF, blood, urine, and other potential sources of infection, confirmed the diagnosis of GPA [6, 7].

The patient was commenced on aspirin and clopidogrel alongside IV heparin. The patient's confusion and immobility had persisted to this point, while her ECGs had returned to normal as had her heart rate and respiratory function. Since she lacked capacity to consent at the time, after discussion with her family, it was decided that more aggressive immunosuppressive treatment would be in her best interest.

**Figure 2:**
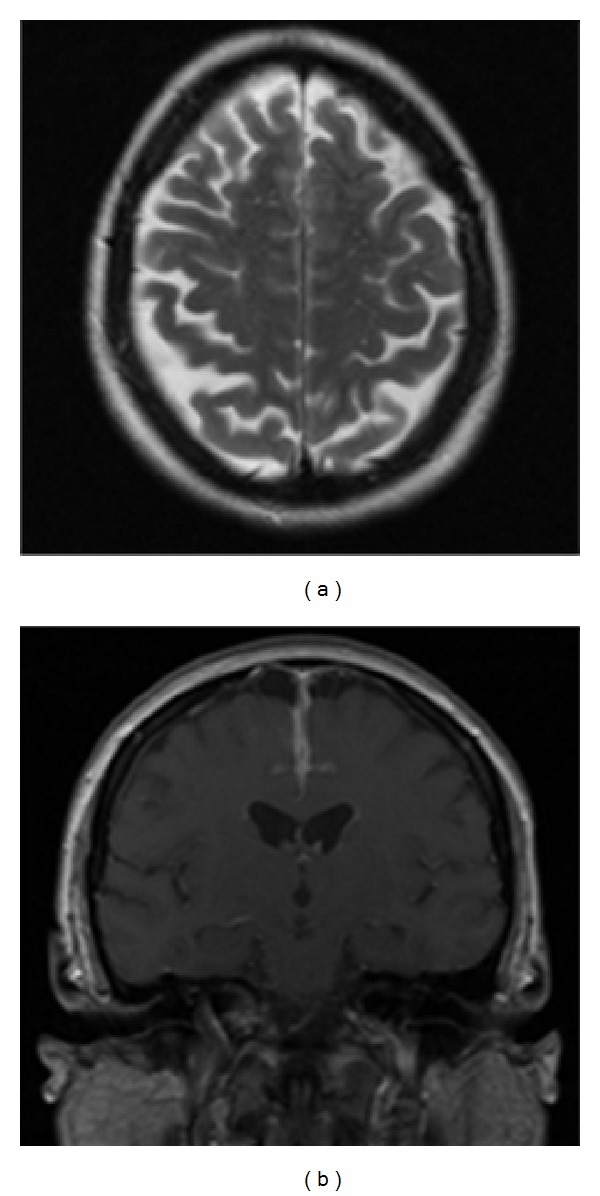
Imaging 5 weeks after presentation, following 3 pulses of methylprednisolone and 2 cycles of IV cyclophosphamide (09/2012). (a) Axial T2 demonstrates substantial resolution of parafalcine oedema. (b) Coronal T1 postcontrast image demonstrates reduction in extent and degree of enhancement in comparison with Figure 1(c).

A rapid clinical response to the intravenous cyclophosphamide therapy was observed [8] and radiological intracranial features demonstrated improvement (Figure 2). Within days of starting cyclophosphamide therapy the patient had a remarkable recovery of cognitive function. She was discharged to outpatient care under rheumatology and neurology with a referral to her local hospital for rehabilitation and physiotherapy. With regard to nonspecific markers of inflammation, initial peripheral blood values were CRP 53 mg/L (peak value 106 mg/L), WBC counts 14.2 × 10^9^/L (peak value 23.5 × 10^9^/L), and neutrophils 10.7 × 10^9^/L (peak value 20.7 × 10^9^/L); haemoglobin remained in the normal range.

**Figure 3:**
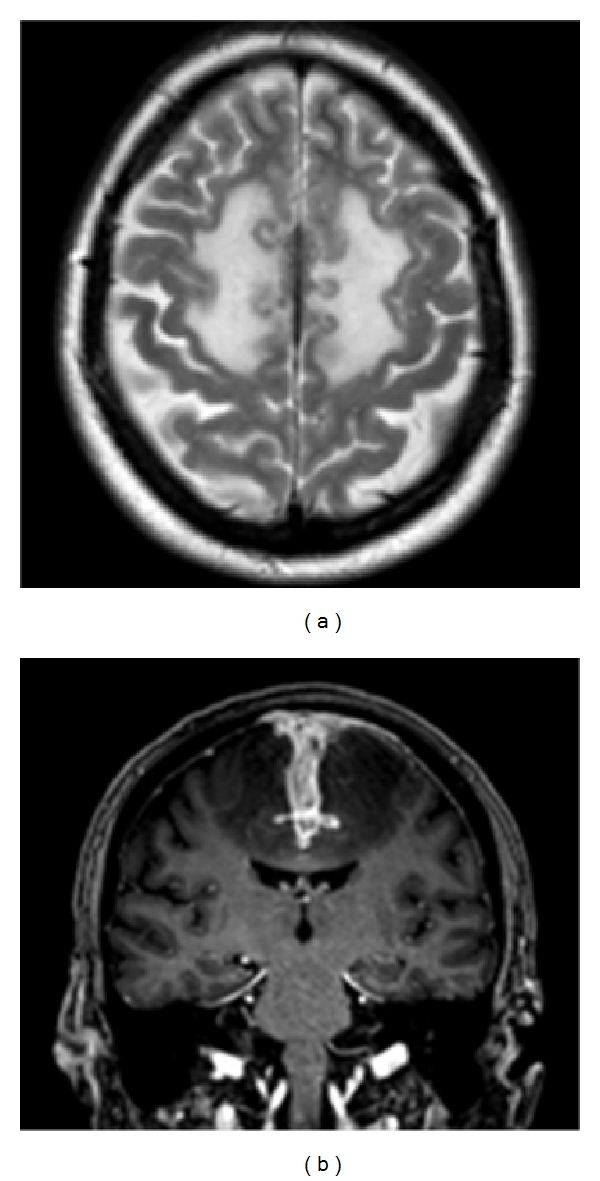
Imaging 9 months after presentation, following 10 cycles of IV cyclophosphamide therapy (05/2013). (a) Axial T2 demonstrates progressive parafalcine oedema, mass effect, and subtle cortical haemorrhage. (b) Coronal T1 postcontrast image demonstrates progressive avid leptomeningeal enhancement, cortical swelling, and avid enhancement of progressively thickened dura.

**Figure 4:**
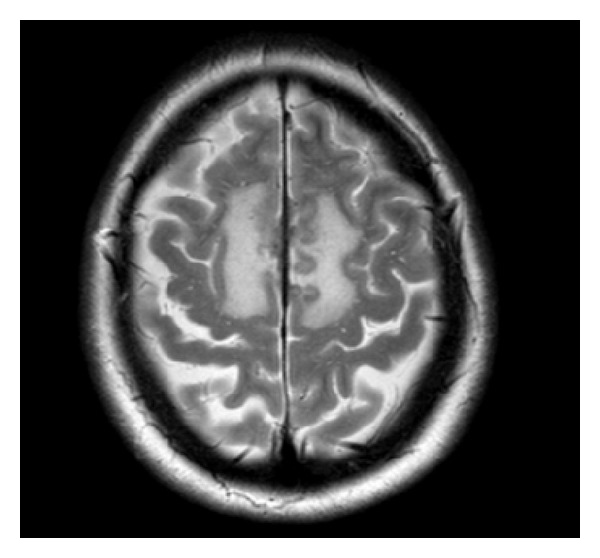
Further axial T2 study 3 months later (08/2013) showing some improvement in extent of T2 abnormality and oedema; dural thickening and enhancement persist.

Treatment was given with intravenous cyclophosphamide pulses (at 15 mg/kg, initially every 2 and then every 4 weeks, without dose alterations as kidney function was normal) with a plan to switch treatment to oral methotrexate after the 10th infusion. Repeat imaging 9 months after presentation was performed shortly after discontinuation of cyclophosphamide and a few weeks before methotrexate could be initiated, a delay caused by a respiratory infection, treated with cotrimoxazole, which interacts with methotrexate. At the time, there was mild cognitive deterioration and imaging demonstrated radiological progression of the underlying disease process (Figure 3). However, other clinical and objective measures of disease activity (including inflammatory markers and BVAS scores [9]) remained stable. Therefore, methotrexate was introduced as planned. Further imaging 12 months after presentation and after 3 months of treatment with methotrexate showed partial improvement in the extent of T2 abnormality and oedema (Figure 4). Further improvement was documented 6 months after the scan presented in Figure 4 (not shown).

## 3. Discussion

GPA is an autoimmune vasculitis of unknown aetiology. It affects the small and medium vessels causing granulomatous lesions. Prior to the development of immunosuppressive therapy, it had an extremely high rate of mortality, but five-year survival is now as high as 90%, albeit with a 40% risk of flare-ups and no cure is available yet [10]. The mainstay of treatment for GPA is cyclophosphamide, a potent nitrogen mustard alkylating agent. There is strong evidence that the benefit of cyclophosphamide therapy outweigh the risks of this medication [8, 11].

The diagnostic criteria from the American College of Rheumatology are particularly useful to identify patients for research purposes [12], but the mainstay of diagnosis is clinical suspicion with positive PR3 c-ANCA tests [2], which are highly specific for GPA. Neurological manifestations are common, as identified in a landmark paper by Nishino et al. in 1993 [3], but cerebritis is the rarest of these manifestations and particularly unusual as the initial means of presentation.

This patient presented with neurological manifestations only and was found to have intracranial lesions with pachymeningitis and cerebritis. In retrospect, the symmetry of the lesions suggested vasculitis, but her normal renal function and lack of involvement of other systems, prior to the CT scan, argued against GPA.

A primary astrocytoma or a meningeal metastasis was among the top initial differential diagnoses, especially with the discovery of an isolated lesion in the lung, but when the CSF was found to be reactive, infection also had to be considered. The absence of pyrexia throughout the admission suggested that if infection was responsible it was unlikely to be a typical organism, with concerns raised about the possibility of tuberculosis, particularly when high dose IV steroids were suggested as a treatment.

With the confirmed anti-PR3 c-ANCA and the initial improvement on IV steroids, the diagnosis of vasculitis became the likeliest explanation for the patient's symptoms, including the lung lesion, now cavitating, and the symmetrical lesions in the brain. Prompt input from rheumatology was very valuable in confirming the diagnosis as an exclusive CNS presentation of GPA is so rare. Pulmonary abnormalities were detected radiologically but were not part of the clinical presentation. This case underscores the need to maintain a thorough differential diagnosis and the benefit of opinions from other specialities early in the investigative process.

In conclusion, the patient was admitted with neurological manifestations of a systemic vasculitis. The diagnosis presented difficulties due to the rarity of this clinical appearance as a primary presentation of GPA. Adequate investigation and prompt input from a number of specialities, including neurology, neurosurgery, rheumatology, cardiology, biochemistry, and immunology, offered a strong basis for commencing chemotherapy and allowed the near complete clinical recovery of a patient who had appeared to be deteriorating unremittingly. A note of caution is warranted in consideration of slower and less marked radiological recovery, indicating that continuing underlying inflammation may lag behind clinical improvement and that continued monitoring and immunosuppression are essential. This case report should be useful to highlight an unusual presentation of GPA and the need to consider such condition in the differential diagnosis of encephalopathic presentations of neurological disease.
